# Southern Medical College Commencement

**Published:** 1890-04

**Authors:** 


### SOUTHERN MEDICAL COLLEGE COMMENCEMENT.

The Southern Medical Cellege held its eleventh Annual Commencement on the night of March 5th.

The exercises were opened with prayer by Prof. Lane of the Technological School. The Dean then read his Report showing a very favorable condition of the School, the total number of students of the two Departments of Medicine and Dentistry aggregating 147.

The following gentlemen were presented as entitled to Diplomas —to-wit:

IN MEDICINE.

R. E. Adair, Georgia; F. L. Adams, Georgia; J. M. Bates, Georgia; G. W. Battle, Georgia; F. M. Broach, Alabama; G. V. Bush, Georgia; J. E. Cole, South Carolina; W. B. Cox, South Carolina; M. Z. Crist, Kentucky; A. R. Davis, Alabama; J. L. Dedge, Georgia; A. J. Dennis,Alabama; G. .L Ezzard, Georgia; J. T. A. Gaines, Georgia; N. C. Goss, Georgia; C. S. Harris, Georgia; J. P. Hunter, Georgia; H. Linley, Texas; J. A. Link, Georgia; H. M. McGee, Georgia; W. S. McHan, Georgia; W. M. Mundy, Tennessee; G. W. Pierce, Georgia; C. L. Purdy, Virginia; M. A. Purse, Georgia; M. R. Stewart, Georgia; M. L. Stipe, Texas; W. L. Thompson, Georgia; T. L. Treadaway, Texas; W. G. Turner,Georgia; G. M. Vincent, Florida; W. S. Ward, Alabama; W. B, Watkins, Georgia; N. A. Williams, Florida.

IN DENTISTRY.

H. J. Arbeely, Syria; T F Brannen, Georgia; W F Blassengame, Georgia; J C Cato, Alabama; C K Chapman, Georgia; J R Dedge, Georgia; A L GriffinL Georgia; J T Gordon; C W Hendry, Georgia; E Y Thomas, Georgia; J H Fandry, Louisiana; T J McIver, Alabama. C H Parrith, Georgia; D Roberts, Georgia; W S Simmons, Georgia.

The graduates presented themselves as their names were called by the respective Deans of the two Departments—Dr. W. P. Nicolson on the part of the Medical Faculty and Dr. Wm. Crenshaw on the part of the Dental Faculty.

The Degrees were conferred by Dr. T. S. Powell, President of the institution, who also delivered the charge in an able address, which was replete with so und doctrine and good advice.

Dr. Wm. Crenshaw spoke in high terms of the success and efficiency of the Dental Department of the Institution.

Dr. Wm. A. Purse of Savannah, Ga., delivered the valedictory address on the part of the Medical class. The address was in all respects an unusually fine one, ornate and beautiful in style and evincing much culture and scientific research, and was well delivered.

The Dental valedictorian was Dr. J. C. Cato, of Alabama. His address was a good one and well delivered.

The Annual Oration was made by the Rev. Boling Sasnett. The speaker was introduced by the Rev. Mr. Lane. His address was a very condensed and interesting outline of the history of Medicine from the time of Hippocrates to the present time. In the concluding part he made some eloquent and interesting admonitory remarks to the class. His address was well delivered and warmly received.

The exercises were closed with the delivery of the prizes—which were conferred as follows:

First Honor, a gold medal, to Dr. T. L. Treadaway of Texas.

Second Honor—an amputating case, to Dr. M. A. Purse, of Georgia.

Third Honor—a pocket case of instruments to Dr. J. M. Bates, of Georgia.

In the Dental Department, the First Honor was awarded to Dr. E. G. Thomas, of Georgia. Second Honor to Dr. C. H. Parish, of Alabama. Third Honor to Dr. H. J. Arbeely, of Syria.

An individual prize by Dr. Elkin, Demonstrator of Anatomy, was conferred on Dr. Chas. H. Finley, for greatest proficiency in dissection.

The Demonstrator's Prize in Dentistry was conferred by Dr. Thos. Crenshaw, on Dr. J. H. Landry.

The exercises were interspersed with music, and altogether the occasion was highly interesting and entertaining.

---

